# Advancing the Noninvasive Diagnosis of Cytomegalovirus Gastrointestinal Disease: Perspectives on Plasma Cytomegalovirus DNA and Clinical Prediction Models

**DOI:** 10.5152/tjg.2026.26551

**Published:** 2026-09-15

**Authors:** Selçuk Candan

**Affiliations:** Department of Gastroenterology, Kanuni Sultan Süleyman Training and Research Hospital, University of Health Sciences, İstanbul, Türkiye

Dear Editor,

I read with great interest the recent article by Karahan et al, entitled “Is It Possible to Determine Plasma Cytomegalovirus DNA Cut-off and Develop a Scoring System to Predict Cytomegalovirus Gastrointestinal Disease?”^1^ The authors should be congratulated for addressing an important and clinically relevant challenge in gastroenterology and infectious diseases. Cytomegalovirus gastrointestinal disease (CMV-GID) remains a potentially life-threatening condition, particularly among immunocompromised individuals, in whom delayed diagnosis may result in substantial morbidity and mortality.^2^ Although histopathological examination remains the diagnostic gold standard, there is an increasing need for reliable, noninvasive adjunctive tools to facilitate the early recognition of CMV-GID in routine clinical practice.^2^ In this context, the authors provide valuable evidence by identifying an overall plasma cytomegalovirus DNA (CMV DNA) threshold of 272 copies/mL and proposing a practical clinical scoring system incorporating age, systemic steroid use, viral load, and characteristic endoscopic findings such as ulcers and pancolitis. This approach may assist clinicians in identifying patients at increased risk of CMV-GID before definitive pathological confirmation.^1^ We believe that this study represents an important step toward improving risk stratification and promoting a more individualized diagnostic approach for patients with suspected CMV-GID.

One of the most valuable aspects of this study is its emphasis on the ongoing shift in CMV-GID diagnosis from reliance on a single diagnostic modality toward a more integrated diagnostic strategy. In routine clinical practice, therapeutic decisions often must be made before definitive pathological confirmation is available, particularly in patients who are critically ill or profoundly immunocompromised.^3^ In this context, combining plasma CMV DNA levels with readily available clinical and endoscopic findings, as proposed by the authors, represents a practical and clinically meaningful approach to early risk stratification.^3^ Importantly, plasma CMV DNA alone cannot reliably establish tissue-invasive gastrointestinal disease and should not be considered a substitute for histopathological confirmation. Rather, plasma viral load and predictive models should be regarded as complementary tools that support risk stratification and clinical judgment while definitive pathological evaluation is pursued. An important question, however, is how patients classified as high risk by such models should be managed in routine clinical practice. At present, the most appropriate role of the proposed model may be to increase diagnostic vigilance and facilitate timely endoscopic and histopathological evaluation in high-risk patients rather than to directly guide therapeutic decisions. Such an approach may be particularly valuable in settings where prompt histopathological assessment is not readily feasible.^3^

The findings reported by Karahan et al^1^ also provide a valuable foundation for future research aimed at further refining noninvasive diagnostic strategies for CMV-GID. As diagnostic approaches continue to evolve, integrating quantitative plasma CMV DNA with additional clinical, endoscopic, and laboratory parameters may further improve diagnostic performance and facilitate more individualized patient management. Furthermore, the marked variation in optimal plasma CMV DNA thresholds across diagnostic subgroups, ranging from 9 copies/mL in patients with inflammatory bowel disease to 298 copies/mL in solid organ transplant recipients, underscores the need for multicenter prospective studies to validate these subgroup-specific thresholds across diverse immunosuppressed populations and laboratory platforms.^1^^,^^4^ This variability also raises an important question as to whether a single plasma CMV DNA threshold can be reliably applied across patient populations with different types and degrees of immunosuppression. Further research may contribute to the development of more standardized and evidence-based diagnostic algorithms while preserving the practicality that makes the proposed model particularly useful in routine clinical practice.

In conclusion, the work by Karahan et al^1^ represents an important contribution to the evolving field of CMV-GID diagnosis. The proposed plasma CMV DNA threshold and practical clinical scoring system are meaningful steps toward improving the early recognition of this challenging condition and informing clinical decision-making. Further investigation in this field may contribute to the development of practical, evidence-based approaches for the diagnosis and management of CMV-GID.

## Data Availability

The data that support the findings of this study are available on request from the corresponding author.
